# Too many lemons to make lemonade? Disentangling mental health during the third wave of COVID-19 infections in Spain

**DOI:** 10.1007/s12144-022-03638-2

**Published:** 2022-10-05

**Authors:** Marta Miragall, Tamara Escrivá-Martínez, Maja Wrzesien, Mª Dolores Vara, Rocío Herrero, Lorena Desdentado, Rosa Mª Baños

**Affiliations:** 1 Department of Personality, Evaluation, and Psychological Treatments, University of Valencia, Avenida Blasco Ibáñez, 21, 46010 Valencia, Spain; 2 CIBER Fisiopatología Obesidad y Nutrición (CIBEROBN), Instituto Carlos III, Madrid, Spain; 3 Instituto Polibienestar, University of Valencia, Av. Blasco Ibáñez 21, Valencia, Spain; 4 Department of Psychology and Sociology, Universidad de Zaragoza, Teruel, Spain

**Keywords:** COVID-19, Positive mental health, Emotional distress, Resilience, Openness to the future, Psychological burden

## Abstract

The study aimed to analyze the longitudinal change in mental health during the third wave of COVID-19 infections in Spain. Negative (e.g., emotional distress) and positive (e.g., positive functioning variables) outcomes were analyzed. Protective factors (e.g., resilience) as predictors of psychological adjustment (i.e., positive mental health, openness to the future, and low burden due to COVID-19) after ten months of the pandemic were also examined. The sample consisted of 164 participants, and self-reported questionnaires were administered at the beginning of the lockdown (March 2020), at the end of the lockdown (June 2020), and during the third wave (January 2021). Linear mixed models showed that individuals’ emotional distress increased, and positive functioning variables (i.e., meaning in life, gratitude, resilience, and life satisfaction) decreased over time, but an increase was observed in some dimensions of posttraumatic growth. Regression analyses showed that resilience scores at all three data collection time points were significant predictors of positive mental health, openness to the future, and burden during the third wave. Mediation analyses showed that positive mental health and openness to the future were mediators of the effect of resilience on burden. The prolonged situation of the COVID-19 crisis had an important impact on positive and negative mental health. However, resilience may help to build up resources that can act as a buffer against adverse psychological effects.

The viral pandemic due to the COVID-19 outbreak became a major worldwide public health crisis. On March 14, 2020, a national lockdown was announced by the Spanish government, and all Spanish citizens were asked to remain physically isolated in their homes and maintain physical distance. These strict restrictions lasted until June 21, 2020. Since then, the pandemic has become a chronic stressor due to several waves of infection that have had serious repercussions for health, political, social, and economic systems around the world.

Numerous studies have shown different negative psychological effects of the COVID-19 outbreak, indicating an increase in post-traumatic stress symptoms, anxiety, and depression, among others (e.g., Brooks et al., 2020; Odriozola-González et al., 2020; Torales et al., 2020, Qiu et al., 2020). The combination of the unpredictable, continuous, and uncontrollable nature of a pandemic (Taha et al., 2014) and the need for social distancing and restrictions (Brooks et al., 2020) has made the situation “magnify our every functional and structural vulnerability” (Horesh & Brown, 2020, p.332). Therefore, the effects of COVID-19 are expected to have a profound and long-lasting impact on mental health and well-being (e.g., Holmes et al., 2020), not only intensifying previously existing mental health issues, but also contributing to the development of new stress-related disorders (Horesh & Brown, 2020).

It has been widely reported that the COVID-19 pandemic has produced a heavy psychological burden on the population (Necho et al., 2021; Robinson et al., 2022). *Psychological burden* due to COVID-19 is defined as the feeling of being overwhelmed, accompanied by negative emotional responses such as anxiety, depression, uncertainty, loss of control, and frustration in daily activities (Brailovskaia et al., 2021). Nevertheless, some authors have also highlighted individuals’ heterogeneous responses in times of the COVID-19 pandemic, suggesting that not everyone experienced burden or negative mental health outcomes (Shevlin et al., 2021). In the same vein, other studies have shown that only a small percentage of people exposed to a specific threatening event develop a clinically significant mental disorder (e.g., Galea et al., 2003). Thus, there is a need to better understand the variations in people’s response patterns (e.g., Bonanno & Mancini, 2012; Vázquez et al., 2021). As this global crisis unfolds, some individuals may experience more severe and long-lasting psychological symptoms, whereas others are able to adapt to the threat, learn something new from it (Shevlin et al., 2021; Taylor, 2019), or even experience positive changes in their psychological functioning (Baños et al., 2021; Tedeschi et al., 2018).

In this regard, it is important to include both negative and positive mental health outcomes when studying the impact of the COVID-19 pandemic (Chen & Bonanno, 2020). According to the Dual Continuum Model of Mental Health (Westerhof & Keyes, 2010), mental illness and positive mental health reflect two distinct continuums rather than the extremes of a single spectrum. Thus, both dimensions should be analyzed. Most studies have focused on examining the adverse effects of COVID-19 on mental health (Necho et al., 2021), but little is known about positive functioning factors that prevent the development of psychopathology and foster the capacity to overcome adversity (Tamiolaki & Kalaitzaki, 2020).

The prolonged duration of the pandemic has provided the opportunity to study the dynamics of positive and negative mental health outcomes as it progresses. The meta-analysis of longitudinal studies by Prati and Mancini (2021) showed that *emotional distress* (ED) increased during the pandemic -although the increase was small-, but the positive functioning variables did not decrease significantly. Robinson et al. (2022) also carried out a meta-analysis that included longitudinal cohort studies analyzing the outcomes before and during the pandemic in 2020. They found a significant -but small - increase in mental health symptoms (especially in depression) early in the pandemic (in March-April 2020). However, in May-June 2020, mental health symptoms were comparable to pre-pandemic levels. Hence, this meta-analysis showed an overall tendency towards psychological adaptation in individuals within a few months of the onset of the pandemic. Nevertheless, few studies have examined the longitudinal changes beyond June 2020. Longitudinal designs are highly relevant for studying temporal changes in positive and negative mental health outcomes as the pandemic progresses.

Although the literature on protective factors during the COVID-19 pandemic is relatively limited, some psychological processes are known to buffer the negative impact of traumatic events. The literature points out that *meaning in life* allows individuals to positively re-evaluate adverse events and mobilize the necessary resources to rediscover themselves, restore their essential assumptive world, and move towards future goals (Updegraff et al., 2008). Along the same lines, *gratitude* in traumatic situations may also be relevant because it leads to greater spiritual depth and helps people to make sense of their lives and view them as a gift (Vernon et al., 2009). Additionally, *life satisfaction* may buffer psychological distress about possible threats (Trzebiński et al., 2020). Thus, some longitudinal studies on gratitude (Fishman, 2020), life satisfaction, or meaning in life (Choi et al., 2021) have shown encouraging protective effects. Finally, the potential growth resulting from the stressor (*post-traumatic growth*, PTG) should also be considered because it is important to explore whether an individual is able to respond adaptively to a stressor while initiating a process of continued growth and gain due to the trauma (Ho, 2016).

In addition, *resilience* stands out as a crucial mechanism in experiencing psychological adjustment during the COVID-19 pandemic. This concept is defined as “the personal qualities that enable one to thrive in the face of adversity” (Connor & Davidson, 2003, p.1), and it has been related to a process of negotiation, management, and adaptation to the stressful situation that can foster recovery from adversity (Windle, 2010). According to Davydov et al. (2010), resilience mechanisms “may serve to protect and/or promote mental health, accelerate recovery, and/or mitigate the negative effects of mental’ pathogens’—i.e., stressors” (p. 482). In this regard, Paredes et al. (2021) found that the perception of threat associated with COVID-19 in individuals with a high level of resilience had less impact on future anxiety (i.e., a state of apprehension, uncertainty, fear, worry, and concern about unfavorable changes in one’s personal future, Zaleski, 1996) and, consequently, on subjective well-being. Similarly, Riehm et al. (2021) showed that people with high levels of resilience had no changes in ED during lockdown, whereas those with low or normal levels of resilience were twice as likely to experience it. Based on these results, resilience can be considered a potential key factor underlying psychological adjustment (including less ED and higher positive psychological functioning) throughout the COVID-19 crisis.

In this regard, two mechanisms that may be involved in the relationship between resilience and psychological adjustment are openness to the future (as a contrasting response to future anxiety) and positive mental health (as a contrasting response to ED). *Openness to the future*, on the one hand, is defined as “an active cognitive-affective mood state that involves positive expectations about what life may bring, a sense of competence and ability to cope with events, the anticipation, planning, and perseverance to reach an outcome even in the face of adversity, and the acceptance of what cannot be resolved or predicted” (Botella et al., 2018). It has been considered an essential ingredient in coping with negative events that involve a high level of uncertainty, such as COVID-19 (Botella et al., 2018; Vázquez et al., 2021). *Positive mental health*, on the other hand, refers to experiencing general well-being in emotional, psychological, and social dimensions (Lukat et al., 2016). Previous research showed that positive mental health was associated with better psychological adjustment when facing life stressors (Truskauskaite-Kuneviciene et al., 2020), that is, less psychological burden. Therefore, it is conceivable that these two processes -openness to the future and positive mental health- could act as mediator mechanisms of resilience that lead to less psychological burden in the current pandemic situation.

In sum, understanding the evolution of the impact of the COVID-19 crisis on mental health and the changes in ED and positive psychological functioning variables during the pandemic is highly significant (Chen & Bonanno, 2020; Vázquez et al., 2021). To our knowledge, there are no longitudinal studies that include negative and positive mental health outcomes after ten months of the pandemic (i.e., in the third wave of COVID-19 in Spain). Identifying the variables that are most likely to worsen, along with predictors of adjustment and/or maladjustment, could help clinicians to be prepared for future crises and create interventions that target these specific variables. Therefore, the main aim of the present study is to examine the trajectories of ED (i.e., perceived stress, negative affect, anxiety, and depression), positive psychological functioning (i.e., meaning in life, resilience, gratitude, and life satisfaction), and PTG at three different times during the pandemic, and identify protective factors for psychological adjustment. To do so, the study variables were assessed throughout the lockdown period and in the third wave of increased COVID-19 infections in Spain.

The specific objectives of this study are: (1) to identify the changes in ED, positive functioning variables, and PTG at three time points during the pandemic; (2) to identify the protective factors (sociodemographic variables and positive functioning variables) for positive mental health, openness to the future, and burden due to COVID-19 in the third wave; and (3) to analyze whether resilience at all three time points predicts burden due to COVID-19 through the mediating effect of positive mental health and openness to the future. Based on the literature, our first hypothesis is that the positive functioning variables will decrease in the third wave of infections (vs. the beginning and end of lockdown), whereas ED will increase. Regarding PTG, based on the studies that indicate the possibility of PTG during chronic adverse situations, we expect an increase in its dimensions. Our second hypothesis is that positive functioning variables, and especially resilience, will be significant predictors of greater positive mental health and openness to the future, as well as lower burden due to COVID-19. Finally, our third hypothesis is that higher positive mental health and openness to the future will be key mediators of the effect of high resilience on the burden due to the COVID-19 crisis 10 months after the first lockdown began in Spain.

## Method

### Participants

The sample consisted of 164 participants. Three inclusion criteria were considered: being over 18 years old; living in Spain at the time of the lockdown and during the third wave; and being a participant enrolled in the study that started on March 21, 2020 (Miragall et al., 2021). Hence, 438 participants (78.3% women; *M* = 35.68; *SD* = 13.19) who completed the first assessment were asked to collaborate in the third wave of the study. There were no exclusion criteria.

Each participant answered the survey three times in a period of 10 months. Sample size decreased in Time 2: Times 1 and 3 included 164 participants (78.1% female, *M*_*age*_=35.40, *SD*_*age*_=13.06); and Time 2 had 110 participants (83.2% female, *M*_*age*_=36.48, *SD*_*age*_=13.40). Sociodemographic variables are described in Table 1.

**Table 1:** Sociodemographic information about the sample

	Times 1 & 3 *N =* 164	Time 2 *N =* 110
**Sex (%women)**	81.1%	80.9%
**Age (years)** ***M (SD)***	36.95 (14.10)	37.87 (14.13)
18–24 25–35 36–50 > 50	24.4% 31.15% 23.2% 21.3%	20.0% 33.6% 23.6% 22.7%
**Diagnosis of Mental illness (% yes)**	6.7%	8.2%
**Diagnosis of Chronic disease (% yes)**	20.1%	24.5%
**Marital status** Single In a relationship Married Divorced/Separated Widowed Other	23.8% 35.4% 29.9% 7.9% 1.2% 1.8%	21.8% 36.4% 30.9% 8.2% 1.8% 0.9%
**Monetary income** Below the mean At the mean Above the mean	35.4% 51.2% 13.4%	33.6% 51.8% 14.5%
**Employment situation** Employee (permanent job) Employee (temporal job) Freelancer Job seeker Student Other	36.0% (36.6%) ^a^ 18.3% (24.4%) ^a^ 3.7% (4.9%) ^a^ 4.3% (3.7%) ^a^ 26.8% (18.9%) ^a^ 11.0% (11.6%) ^a^	38.2% 19.1% 2.7% 3.6% 24.5% 11.8%
**Diagnosis of coronavirus (% yes)**	1.2% (10.4%) ^a^	2.7%
**Relative or close person diagnosed with coronavirus (% yes)**	14.6% (61.6%) ^a^	41.8%

*Note.*^a^ Percentages between brackets refer to Time 3

The informed consent was signed by the participants before answering the questionnaires. The study was conducted following the Declaration of Helsinki and approved by the ethical committee of the University of Valencia (Spain) (register number: 1,593,681,212,393).

### Measures

#### Sociodemographic characteristics

Participants included information about their sex, age, diagnoses of chronic and mental illnesses, marital status, income level, and employment status. In addition, they reported whether they and/or a person close to them had been diagnosed with COVID-19.

#### Positive functioning measures

**Presence and search for meaning.** The Meaning in Life Questionnaire (Steger et al., 2006) consists of 10 items that assess two dimensions of meaning in life: (1) presence of meaning (MLQ-P) and (2) search for meaning (MLQ-S). Individuals rated each item on a seven-point Likert scale. Internal consistency for the MLQ-P ranged between α = 0.90 and α = 0.91, and for the MLQ-S between α = 0.93 and α = 0.96, over time.

**Gratitude.** The Gratitude Questionnaire-6 (GQ-6, Magallares et al., 2018) contains six items that assess the tendency to feel gratitude in daily life. Individuals rated each item on a seven-point Likert scale. Internal consistency ranged between α = 0.72 and α = 0.78 over time.

**Resilience.** The Connor-Davidson Resilience Scale (CD-RISC; Notario-Pacheco et al., 2011) contains 10 items that measure resilience. Individuals rated each item on a five-point Likert-type scale. Internal consistency ranged between α = 0.87 and α = 0.90 over time.

**Life satisfaction.** The Satisfaction with Life Scale (SWLS, Vázquez et al., 2013) contains five items that assess the overall cognitive perception of one’s satisfaction with life. Individuals rated each item on a seven-point Likert scale. Internal consistency ranged between α = 0.87 and α = 0.89 over time.

#### Emotional distress measures

**Perceived stress.** An ad hoc scale (PS) with two items was developed to evaluate the degree to which the individual’s current life is perceived as stressful (“I have felt that I can deal with all the things I should do”; “I have managed the small daily problems”). Individuals rated each item on a five-point Likert scale. Internal consistency ranged between α = 0.72 and α = 0.79 over time.

**Symptoms of depression.** The Patient Health Questionnaire-2 (PHQ-2; Rodríguez-Muñoz et al., 2017) contains two items that explore symptoms of depression. Individuals rated each item on a four-point Likert scale. Internal consistency ranged between α = 0.81 and α = 0.90 over time.

**Negative and positive affect.** The Positive and Negative Affect Schedule (PANAS; López-Gómez et al., 2015) contains 20 items that assess two dimensions: positive affect (PANAS positive) and negative affect (PANAS negative). Individuals rated each item on a five-point Likert scale. Internal consistency for PANAS positive ranged between α = 0.92 and α = 0.95, and for PANAS negative between α = 0.89 and α = 0.91, over time.

**Symptoms of anxiety.** The Generalized Anxiety Disorder Questionnaire-2 (GAD-2; García-Campayo et al., 2012) contains two items that assess symptoms of anxiety. Individuals rated each item on a four-point Likert scale. Internal consistency ranged between α = 0.71 and α = 0.86 over time.

#### Posttraumatic growth (PTG) measure

**Posttraumatic growth**. The short form of the Post-traumatic Growth Inventory (PTGI-SF, Cárdenas et al., 2015) contains 10 items that measure the degree to which people experience positive life changes as a consequence of a major life crisis. Five dimensions of PTG are assessed, with two items in each: relating to others, new possibilities, appreciation of life, personal strength, and spiritual change. Individuals rated each item on a six-point Likert scale. Internal consistency for “new possibilities”, “relating to others”, “personal strength”, and “appreciation of life” ranged from α = 0.71 to α = 0.77, α = 0.80 to α = 0.89, α = 0.82 to α = 0.86, and α = 0.81 to α = 0.86, over time, respectively. Spiritual change had low consistency over time, ranging from α = 0.56 to α = 0.68.

#### Psychological adjustment measures (measured after 10 months of the pandemic)

**Burden due to COVID-19** (Brailovskaia et al., 2021). The attitudes and feelings of psychological burden due to COVID-19 were assessed with six items (e.g., “I am burdened by the current social situation”, “I feel restricted in my everyday life”, “I feel socially isolated”). Individuals rated each item on a seven-point Likert-type scale. This scale showed adequate internal consistency (α = 0.71).

**Positive mental health.** The Positive Mental Health Scale (PMH, Lukat et al., 2016) contains nine items that assess well-being (both subjective and psychological dimensions). Individuals rated each item on a four-point Likert scale. Internal consistency was adequate (α = 0.92).

**Positive view of the future.** The Openness to the future scale (OFS, Botella et al., 2018) contains 10 items that assess positive affect towards the future, taking into account five domains: acceptance, engagement in life and planning, illusion of control, positive future orientation, self-efficacy regarding future plans). Individuals rated each item on a five-point Likert scale. Internal consistency was adequate (α = 0.83).

### Procedure

Participants were invited by email to participate in the follow-up of the study that had begun in March 2020 ( Miragall et al., 2021). They were told that the follow-up was designed to evaluate potential positive psychological factors associated with the lockdown and the third wave of the COVID-19 crisis in Spain.

The survey was completed between March 21 and March 29, 2020 (during the first two weeks of the lockdown in Spain) in Time 1, after June 22, 2020 (after the “new normality” started) in Time 2, and between January 24 and January 31, 2021 (when the third wave of infections started) in Time 3. In Time 3 (third wave), most Spanish communities adopted the following restrictions: closing the perimeter of some communities, a curfew from 10 p.m. to 6 a.m., closing stores and restaurants at 6 p.m., and limiting social meetings in public and private spaces to a maximum of six people.

The web-based tool Qualtrics was used to complete the surveys, which took about 15–20min. Participants answered all the questionnaires listed in the “Measures” section in Times 1–3, except the measures “Burden due to COVID-19”, “Positive Mental Health (PMH)”, and “Openness to the future scale (OFS)”, which were only filled out in Time 3.

### Data analyses

Statistical analyses were conducted using SPSS v.26. First, linear mixed models were performed using the MIXED procedure to analyze the longitudinal change in positive functioning variables, ED, and PTG across the three data collection time points. Specifically, separate models were computed per outcome variable (i.e., MLQ-P, MLQ-S, GQ-6, CD-RISC, SWLS, PS, PHQ-2, GAD-2, PANAS positive, PANAS negative, and PTG dimensions), with time entered as fixed effects and each subject included as random effects. Effect sizes (i.e., the Hedges’ g correction) and their 95% confidence intervals (CIs) were computed for each comparison over time.

Second, nine stepwise multiple regression analyses were computed to determine the predictive role of the sociodemographic (age, sex, marital status, and level of income) and positive functioning variables at Times 1, 2, and 3 in positive mental health, openness to the future, and burden due to COVID-19 in the third wave (Time 3). Specifically, separate models were computed for each outcome variable regressed on the predictors at each data collection time point. To handle the missing data, we used the “pairwise deletion” method. Cohen’s f^2^ = $\frac{{R}^{2}}{1- {R}^{2}}$was calculated to determine whether the effect size of the multiple regression was small (0.02), medium (0.15), or large (0.35) (Cohen, 1988).

Third, three parallel multiple mediation analyses were performed to analyze whether positive mental health and openness to the future mediated the effect of resilience (at each time point) on burden. Specifically, separate models were carried out for resilience at Times 1, 2, and 3. They were computed with the macro for SPSS “PROCESS, version 3.5.3” using Model 4 (Hayes, 2017). To determine the significance of the indirect effects, 95% percentile bootstrap CIs with 5,000 samples were used. These effects were considered significant when the CI did not contain the zero value. When significant indirect effects were found, pairwise comparisons were conducted to determine whether the sizes of the specific indirect effects were significantly different.

## Results

### Changes in positive functioning variables, emotional distress, and posttraumatic growth in Times 1, 2, and 3

Linear mixed models were carried out to test the first hypothesis (i.e., to analyze whether there were increases in ED, decreases in the positive functioning variables, and increases in PTG). Results showed significant main effects of time on the positive functioning variables, ED, and PTG. Table 2 displays the means and standard deviations of the study variables at the three time points, the results of the linear mixed models, and Bonferroni comparisons; and Table 3 shows the effect sizes and their 95% confidence intervals (CIs) for each comparison over time.

**Table 2:** Differences in the study variables over time

		Time 1 *M (SD)* *N = 164*	Time 2 *M (SD)* *N = 110*	Time 3 *M (SD)* *N = 160*	Linear Mixed model	Bonferroni post-hoc comparison
**Positive functioning variables**	**1. Presence of meaning (MLQ-P)**	25.04 (6.77)	25.98 (6.56)	24.65 (6.74)	*F*(2, 263.79) = 0.53, *p =* .590	-
	**2. Search for meaning (MLQ-S)**	18.91 (8.14)	15.61 (8.45)	17.41 (8.49)	*F*(2, 266.31) = 9.67, *p <* .001	T1 > T2 & T3
	**3. Gratitude (GQ-6)**	36.08 (5.14)	34.30 (5.22)	34.87 (5.48)	*F*(2, 273.58) = 7.83, *p <* .001	T1 > T2 & T3
	**4. Resilience (CD-RISC)**	29.66 (6.02)	29.57 (6.63)	28.19 (6.28)	*F*(2, 267.89) = 7.27, *p =* .001	T1 > T3
	**5. Life Satisfaction (SWLS)**	23.84 (6.26)	24.85 (6.19)	23.30 (6.68)	*F*(2, 264.94) = 3.10, *p =* .047	T2 > T3
**Emotional distress**	**6. Perceived Stress (PS)**	1.55 (1.40)	1.51 (1.43)	1.99 (1.33)	*F*(2, 278.23) = 9.74, *p <* .001	T1 & T2 < T3
	**7. Depressive symptoms (PHQ-2)**	1.33 (1.51)	1.18 (1.60)	1.89 (1.81)	*F*(2, 277.87) = 12.78, *p <* .001	T1 & T2 < T3
	**8. Anxiety symptoms (GAD-2)**	2.07 (1.67)	1.80 (1.78)	2.24 (1.60)	*F*(2, 280.92) = 2.63, *p =* .074	-
	**9. Positive affect (PANAS+)**	27.50 (7.71)	30.63 (8.67)	28.41 (9.33)	*F*(2, 278.43) = 6.20, *p =* .002	T1 < T2; T2 > T3
	**10. Negative affect (PANAS -)**	19.50 (6.69)	18.19 (7.24)	20.72 (7.73)	*F*(2, 271.99) = 7.79, *p =* .001	T2 < T3
**PTG dimensions**	**11. New possibilities (PTGI-SF)**	4.82 (2.65)	5.07 (2.85)	5.80 (2.89)	*F*(2, 271.11) = 11.68, *p <* .001	T1 & T2 < T3
	**12. Relating to others (PTGI-SF)**	6.56 (3.02)	5.64 (2.98)	5.83 (2.99)	*F*(2, 268.79) = 8.63, *p <* .001	T1 > T2 & T3
	**13. Personal strength (PTGI-SF)**	5.43 (3.14)	6.30 (3.19)	6.34 (3.10)	*F*(2, 270.00) = 9.61, *p <* .001	T1 < T2 & T3
	**14. Appreciation of life (PTGI-SF)**	6.21 (3.02)	5.75 (2.96)	6.74 (3.06)	*F*(2, 273.01) = 5.43, *p =* .005	T2 < T3
	**15. Spiritual change (PTGI-SF)**	3.65 (2.26)	3.26 (2.18)	3.64 (2.25)	*F*(2,265.78) = 2.95, *p =* .054	-

*Notes.* MLQ = The Meaning in Life Questionnaire; GQ-6 = The Gratitude Questionnaire-6; CD-RISC = The Connor-Davidson Resilience Scale; SWLS = The Satisfaction with Life Scale; PS = Perceived Stress; PHQ-2 = The Patient Health Questionnaire-2; GAD – 2 = The Generalized Anxiety Disorder Questionnaire-2; PANAS = Positive and Negative Affect Schedule; PTGI-SF = short form of the Posttraumatic Growth Inventory

**Table 3:** Effect sizes and 95% confidence intervals (CIs) for each comparison over time

		Time 1 vs. Time 2 *Hedges’ g, 95% CI*	Time 2 vs. Time 3 *Hedges’ g, 95% CI*	Time 1 vs. Time 3 *Hedges’ g, 95% CI*
**Positive functioning variables**	**1. Presence of meaning (MLQ-P)**	-0.14, [-0.28, 0.00]	0.20, [0.05, 0.35]	0.06, [-0.08, 0.20]
	**2. Search for meaning (MLQ-S)**	0.39, [0.23, 0.57]	-0.21, [-0.32, − 0.10]	0.18, [0.03, 0.33]
	**3. Gratitude (GQ-6)**	0.34, [0.18, 0.51]	-0.11, [-0.29, 0.07]	0.23, [0.06, 0.40]
	**4. Resilience (CD-RISC)**	0.01, [-0.12, 0.15]	0.21, [0.06, 0.36]	0.24, [0.11, 0.37]
	**5. Life Satisfaction (SWLS)**	-0.16, [-0.28, − 0.04]	0.24, [0.09, 0.39]	0.08, [-0.04, 0.21]
**Emotional distress**	**6. Perceived Stress (PS)**	0.03, [-0.15, 0.21]	-0.35, [-0.55, − 0.14]	-0.32, [-0.49, − 0.16]
	**7. Depressive symptoms (PHQ-2)**	0.10, [-0.07, 0.26]	-0.41, [-0.64, − 0.19]	-0.33, [-0.49, − 0.18]
	**8. Anxiety symptoms (GAD-2)**	0.16, [-0.03, 0.34]	-0.26, [-0.46, − 0.05]	-0.10, [-0.31, 0.10]
	**9. Positive affect (PANAS+)**	-0.38, [-0.59, − 0.17]	0.24, [0.06, 0.44]	-0.11, [-0.27, 0.06]
	**10. Negative affect (PANAS -)**	-0.19, [0.02, 0.35]	-0.34, [-0.52, − 0.16]	-0.17, [-0.32, − 0.02]
**PTG dimensions**	**11. New possibilities (PTGI-SF)**	-0.09, [-0.28, 0.10]	-0.25, [-0.40, − 0.11]	-0.35, [-0.52, − 0.18]
	**12. Relating to others (PTGI-SF)**	0.30, [0.13, 0.48]	-0.06, [-0.20, 0.07]	0.24, [0.08, 0.40]
	**13. Personal strength (PTGI-SF)**	-0.27, [-0.45, − 0.10]	-0.01, [-0.16, 0.14]	-0.29, [-0.45, − 0.13]
	**14. Appreciation of life (PTGI-SF)**	0.15, [-0.04, 0.34]	-0.33, [-0.50, − 0.15]	-0.17, [-0.33, − 0.01]
	**15. Spiritual change (PTGI-SF)**	0.17, [0.01, 0.34]	-0.17, [-0.29, − 0.05]	0.00, [-0.14, 0.15]

*Notes.* MLQ = The Meaning in Life Questionnaire; GQ-6 = The Gratitude Questionnaire-6; CD-RISC = The Connor-Davidson Resilience Scale; SWLS = The Satisfaction with Life Scale; PS = Perceived Stress; PHQ-2 = The Patient Health Questionnaire-2; GAD – 2 = The Generalized Anxiety Disorder Questionnaire-2; PANAS = Positive and Negative Affect Schedule; PTGI-SF = short form of the Posttraumatic Growth Inventory

Regarding the positive functioning variables, scores on search for meaning and gratitude in Times 2 and 3 were significantly lower than in Time (1) Moreover, resilience and life satisfaction were significantly lower in Time 3 than in Times 1 and (2) Non-significant differences were found for presence of meaning, which remained stable (*p* > .05).

Regarding ED, scores on perceived stress and symptoms of depression were significantly higher in Time 3 than in Times 1 and 2. Negative affect was significantly higher in Time 3 than in Time 2. In contrast, positive affect was significantly lower in Time 3 than in Time 2, and significantly lower in Time 1 than in Time 2. Non-significant differences were found for symptoms of anxiety (*p* > .05).

With regard to the PTG dimensions, scores on new possibilities were higher in Time 3 than in Times 1 and 2. Appreciation of life was significantly higher in Time 3 than in Time 2. Personal strength was significantly higher in Times 2 and 3 than in Time 1. In contrast, scores on relating to others were significantly higher in Time 1 than in Times 2 and 3. Non-significant differences were found for the dimension of spirituality (*p* > .05).

### Protective factors of positive mental health, openness to the future, and psychological burden in the third wave of COVID-19 (Time 3)

To check the second hypothesis (i.e., whether the positive functioning variables were predictors of greater positive mental health, more openness to the future, and lower burden), nine stepwise multiple regression analyses were carried out. Table 4 shows the regression analyses predicting positive mental health, openness to the future, and psychological burden in the third wave (Time 3), based on sociodemographic and positive functioning variables (Times 1, 2, and 3). According to established guidelines (Bowerman & O’Connell, 1990; Myers & Broyles, 2000), no problems with multicollinearity were found, given that the Variance Inflation Factor ranged from 1.00 to 1.79 for all the regressions tested.

**Table 4:** Models for sociodemographic variables and positive functioning variables as predictors of positive mental health, openness to future, and burden in the third wave (*N* = 164)

Outcomes	Predictors	R	Adjusted R^2^	R^2^ Change	B	SE	β	t
**Time 1**								
**Positive mental health (PMH)**	Constant				9.34	2.17		4.31***
	Life satisfaction	0.48	0.23	0.23	0.31	0.07	0.34	4.44***
	Resilience	0.54	0.28	0.06	0.24	0.07	0.25	3.20**
	Sex	0.56	0.30	0.02	2.30	1.01	0.16	2.29*
**Openness to the future (OFS)**	Constant				6.37	1.58		4.04***
	Resilience	0.50	0.24	0.25	0.22	0.06	0.33	4.10***
	Presence of meaning Sex	0.55 0.56	0.29 0.30	0.05 0.02	0.17 1.57	0.05 0.73	0.28 0.15	3.54*** 2.13*
**Burden**	Constant				61.44	3.85		15.96***
	Resilience Sex	0.33 0.37	0.10 0.13	0.10 0.03	-0.46 -4.37	0.12 1.85	− 0.29 − 0.18	-3.87*** -2.36*
**Time 2**								
**Positive mental health (PMH)**	Constant				10.06	2.19		4.59***
	Resilience	0.55	0.29	0.30	0.32	0.08	0.37	3.87***
	Life satisfaction	0.60	0.35	0.06	0.28	0.09	0.30	3.16**
**Openness to the future (OFS)**	Constant				7.64	1.46		5.25***
	Resilience	0.62	0.38	0.39	0.39	0.05	0.62	8.07***
**Burden**	Constant				61.02	3.79		16.11***
	Resilience	0.44	0.19	0.19	-0.62	0.13	− 0.44	-4.96***
**Time 3**								
**Positive mental health (PMH)**	Constant				2.24	2.11		1.06
	Life satisfaction	0.71	0.50	0.50	0.36	0.06	0.42	6.19***
	Resilience	0.77	0.59	0.09	0.31	0.06	0.34	5.29***
	Gratitude	0.78	0.60	0.02	0.15	0.06	0.15	2.52*
	Sex	0.79	0.61	0.01	1.58	0.77	0.11	2.06*
**Openness to the future (OFS)**	Constant				3.12	1.70		1.83
	Resilience	0.65	0.42	0.42	0.38	0.04	0.57	8.78***
	Gratitude	0.68	0.45	0.04	0.16	0.05	0.21	3.17**
**Burden**	Constant				62.90	3.06		20.45***
	Resilience	0.48	0.23	0.23	-0.72	0.11	− 0.48	-6.73***

*Notes.* PMH = Positive Mental Health Scale; OFS = Openness to the Future Scale. * *p* < .05; ** *p* < .01; *** *p* < .001

*Notes.* *p < .05; **p < .01; ***p < .001. All coefficients represent regression coefficients (unstandardized) and standard errors (in parenthesis). Continuous lines imply significant effects, whereas discontinuous lines imply nonsignificant effects. Due to missing values, mediation analyses for Resilience at Times 1, 2, and 3 as predictors were carried out with *n* = 156, *n* = 105, and *n* = 160, respectively. CD-RISC = Connor-Davidson Resilience Scale; PMH = Positive Mental Health Scale; OFS = Openness to the Future Scale

#### Sociodemographic variables and positive functioning variables in Time 1 as predictors

Regarding the model predicting *positive mental health* (PMH), scores on life satisfaction, resilience, and sex (i.e., men) were significant positive predictors, *F*(3,156) = 23.24, *p* < .001, *f*^2^ = 0.46, accounting for 30.0% of the variance. With regard to the model predicting *openness to the future*, resilience, presence of meaning in life, and sex (i.e., men) were significant positive predictors, *F*(3,155) = 23.56, *p* < .001, *f*^2^ = 0.46, accounting for 30.4% of the variance. In the case of the model predicting *psychological burden*, resilience and sex (i.e., men) were significant negative predictor, *F*(2,156) = 12.32, *p* < .001, *f*^2^ = 0.16, explaining 12.7% of the variance.

#### Sociodemographic variables and positive functioning variables in Time 2 as predictors

 Regarding the model predicting *positive mental health* (PMH), resilience and life satisfaction were positive significant predictors, *F*(2, 105) = 29.18, *p* < .001, *f*^2^ = 0.57, explaining 34.9% of the variance. With regard to the regression model for *openness to the future*, resilience was the only significant positive predictor, *F*(1,104) = 65.10, *p* < .001, *f*^2^ = 0.63, explaining 38.1% of the variance. In the case of the model predicting *psychological burden*, resilience was the only negative significant predictor, *F*(1, 104) = 24.56, *p* < .001, *f*^2^ = 0.24, explaining 18.5% of the variance.

#### Sociodemographic variables and positive functioning variables in Time 3 as predictors

Regarding the model predicting *positive mental health* (PMH), satisfaction with life, resilience, gratitude, and sex (i.e., men) were significant positive predictors, *F*(4, 152) = 60.25, *p* < .001, *f*^2^ = 1.564, explaining 60.9% of the variance. With regard to the model predicting *openness to the future*, resilience and gratitude were significant positive predictors, *F*(2, 152) = 62.62, *p* < .001, *f*^2^ = 0.83, accounting for 44.8% of the variance. In the case of the model predicting *psychological burden*, resilience was the only significant negative predictor, *F* (1, 152) = 45.28, *p* < .001, *f*^2^ = 0.30, explaining 22.6% of the variance.

Overall, it should be noted that age, income level, marital status, and the search for meaning were not significant predictors in any model (*p* > .05).

### Resilience at Times 1, 2, and 3 as predictors of psychological burden in the third wave (Time 3), mediated by positive mental health and openness to the future

**Fig. 1:**
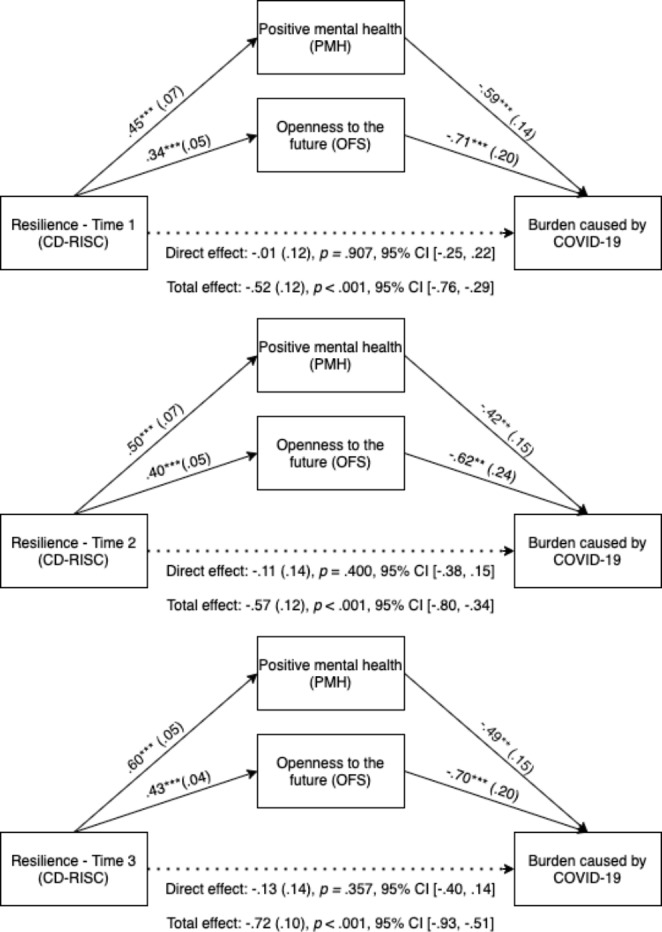
Positive mental health and openness to the future as mediators between resilience and burden due to COVID-19

Three parallel mediation analyses were carried out to test the third hypothesis (i.e., that positive mental health and openness to the future would mediate the effect of resilience on psychological burden). Figure 1 displays the coefficients and standard errors of the parallel multiple mediation models tested to analyze whether positive mental health and openness to the future mediated the effect of resilience at Times 1, 2, and 3 on burden in the third wave (Time 3), as well as the CIs for the direct effects.

The total indirect effects were significant for the three models (Model for Resilience at Time 1: *b* = − 0.51, *SE* = 0.09, 95% CI [-0.69, − 0.35]; Model for Resilience at Time 2: *b* = − 0.46, *SE* = 0.11, 95% CI [-0.69, − 0.26]; Model for Resilience at Time 3: *b* = − 0.59, *SE* = 0.11, 95% CI [-0.81, − 0.39]), which means that positive mental health and openness to the future collectively mediated the relationship between resilience (at Times 1, 2, and 3) and psychological burden at Time 3. Moreover, the specific indirect effects indicated that scores on both positive mental health and openness to the future were mediators of the effect of resilience at Times 1, 2, and 3 on psychological burden. Specifically, individuals who scored higher on resilience had higher scores on both positive mental health and openness to the future, which, in turn, led to a lower burden in the third wave. No significant differences were found in the size of the specific indirect effect of positive mental health or openness to the future.

However, the direct effects of resilience at Times 1, 2, and 3 on burden were not significant (i.e., when the effects of the mediators were maintained constant, the effect of resilience on burden was not significant). The overall models for resilience at Times 1, 2, and 3, including all the variables (the predictor and mediators), were significant (Model for Resilience at Time 1: *F*(3, 152) = 31.56, *p* < .001; Model for Resilience at Time 2: *F*(3, 101) = 19.59, *p* < .001; Model for Resilience at Time 3: *F*(3, 156) = 31.31, *p* < .001), and explained 38.4%, 36.8%, and 37.6% of the variance in burden, respectively.

## Discussion

It is crucial to understand the impact of the life-threatening pandemic on mental health. However, studies that examine individuals’ responses longitudinally -beyond June 2020-, and the role of protective factors when dealing with this chronic adverse event, are still relatively scarce. This paper aimed to extend the study of the long-lasting effects of the COVID-19 pandemic on negative and positive mental health outcomes. To do so, we analyzed: (1) the differences in positive functioning variables, ED, and PTG across three time points (i.e., March 2020, June 2020, and January 2021); (2) the protective factors of psychological adjustment; and (3) the mediational role of positive mental health and openness to the future in the effect of resilience on psychological burden in the third wave of infections.

Regarding the psychological changes during the ten months of the pandemic, results showed that the COVID-19 pandemic had a significantly negative impact on most of the indicators of psychological adjustment. Overall, all the positive functioning variables significantly decreased in the third wave (compared to the beginning of the pandemic and the end of the lockdown) -except the presence of meaning in life, which remained stable-. Similarly, all the ED variables significantly increased during the pandemic (except anxiety symptoms, which remained stable), with effect sizes ranging from small to small-moderate. Nevertheless, some promising changes in PTG were found, given that the majority of its facets (new possibilities, appreciation of life, personal strength) significantly increased during the pandemic, with small or small-moderate effect sizes. The “spirituality” facet of PTG remained stable, and only the “relating to others” facet significantly decreased in the third wave of infections (compared to the other two time points).

Therefore, our first hypothesis was partially supported. Results suggest that prolonged pandemic circumstances have a small -but significant- negative impact on mental health, which is consistent with the meta-analysis by Prati and Mancini (2021) that included longitudinal studies. Nevertheless, the results point out that these adverse circumstances also have a positive impact in terms of PTG. These findings are congruent with other studies that have shown that both ED indicators and PTG can coexist after different types of adverse events (e.g., Zięba et al., 2019), including COVID-19 circumstances (Vázquez et al., 2021; Waters et al., 2021). Indeed, Waters and her colleagues (2021) found that PTG and ED have a “building” type of interaction, where an individual can use an adverse event in a transformational way to establish new attitudes and behaviors that can promote greater mental health in the future.

An interesting finding is related to the decrease in the “relating to others” dimension after ten months of pandemic. Along these lines, other studies have also shown a significant decrease in the size and density of relational networks after the pandemic outbreak (e.g., Kovacs et al., 2021). One possible explanation is that the imposed restriction of social distancing significantly decreased social networks and was associated with greater feelings of loneliness (Kovacs et al., 2021). Given the secondary health effects of increased loneliness (e.g., Hawkley & Cacioppo, 2010), this important factor should be addressed when facing similar challenges in the future.

Regarding our second hypothesis, we found support for the predictive buffering role of positive functioning variables in psychological adjustment outcomes during the third wave of infections. First, in the case of *positive mental health*, higher levels of resilience and life satisfaction were important significant predictors, explaining a large percentage of the variance. Moreover, high levels of gratitude and being a man also made a small -but significant- contribution to the explained variance in positive mental health. Second, with regard to *openness to the future*, resilience also accounted for an important percentage of the explained variance, but the presence of meaning in life, gratitude, and being a man also had a small -but significant-contribution. Third, in the case of *psychological burden*, resilience was the only significant predictor of this indicator of psychological adjustment (although being a man had also a small significant contribution). In sum, our findings emphasize the importance of resilience during the pandemic crisis because it was the only constant predictor of the three indicators of psychological adjustment in the third wave of infections.

Our third hypothesis was fully supported. We found that positive mental health and openness to the future mediated the relationship between resilience and psychological burden. Greater resilience (at all three time points) was related to higher positive mental health and openness to the future, which in turn led to less psychological burden in the third wave, explaining 36–38% of the variance. Thus, our results suggest that resilience produced a “cascade” of positive mental health in cognitive, emotional, and social domains, along with a positive view of the future, which buffered the possibility of experiencing psychological burden.

Therefore, positive mental health and openness to the future arise as two key mechanisms for dealing with “pandemic fatigue”. The mediational effect of positive mental health found in this study coincides with several studies that have proposed it as a protective factor against highly adverse psychological consequences (e.g., Brailovskaia et al., 2020). The mediating role of openness to the future is also congruent with other studies that showed that focusing on future goals may enhance well-being after traumatic events, such as 9/11 (e.g., Holman & Silver, 2005). Regarding openness to the future, only a few studies have included this variable as a mechanism contributing to psychological outcomes during COVID-19, and the results have been mixed. For example, Vázquez and his colleagues (2021) showed that, although openness to the future had a significant effect on PTG, the impact on posttraumatic stress symptoms was lacking. Similarly, Valiente and her colleagues (2021) showed no significant effects of openness to the future on psychological symptoms over time. Hence, more longitudinal studies are needed to disentangle the potential role of cultivating a positive view of the future during chronic stressors involving an uncertain outcome.

This study has several limitations that should be mentioned. First, the sample size in the follow-up measurement was relatively small (i.e., 37.4% of the initial sample). Second, the percentage of females and males was not balanced. These issues affect the sample’s representativeness, limiting the generalization of the results to other populations. Future studies should consider clinical samples and examine the role of gender in mental health during the current pandemic (e.g., Yildirim & Eslen-Ziya, 2021). Third, PTG was evaluated when the adverse event was still ongoing. Thus, the results may only represent the initial coping strategy, which might change, given its dynamic nature. Indeed, PTG can be seen as a construct that evolves from an initial coping strategy to an enduring positive personality trait and/or philosophical view of the world (Vázquez et al., 2021), and building up these positive responses after facing adverse situations takes time.

Despite the limitations mentioned, the current study offers several valuable clinical insights. Our findings indicate that the prolonged pandemic crisis significantly impacted positive and negative mental health, and that resilience helped individuals to build up resources to act as a buffer against the adverse psychological effects. In this regard, tailored interventions to foster resilience, while considering the two mediators that stood out in our findings (i.e., positive mental health and openness to the future), may be useful for buffering the effects of a prolonged stressor such as a pandemic. According to the Adverse Childhood Experience framework (Sciaraffa et al., 2018), three categories of “core protective systems” to cultivate resilience can be identified, related to individual capacities, interpersonal relationships, and the role of community. From this perspective, psychological interventions should consider these three specific factors to accurately target resilience and, ultimately, promote psychological adjustment. Moreover, interventions targeting interpersonal relationships, in an online or face-to-face format, could decrease the sense of loneliness that seems to be especially affected as the pandemic progresses (Pai & Vella, 2021). Finally, providing interventions (e.g., trauma-informed education), enhancing policy systems (Mortensen & Barnett, 2016), or using the protective role of community (including faith, traditions, and cultural processes) (Sciaraffa et al., 2018) might be other ways to foster resilience from a socioecological perspective (Zhang et al., 2022).

During this unprecedented worldwide crisis, investigating the psychological processes through which individuals are sustained and strengthened provides a broader view of the way society is dealing with this highly challenging experience. According to Buheji et al. (2020), “managing COVID-19 is more than hand washing and social distancing; instead, it is a story between hope and despair” (p. 9). Therefore, efforts to develop strategies to promote this hope through different clinical interventions are urgently needed (e.g., Holmes et al., 2020). Future studies should examine the effects of interventions targeting resilience on individuals’ psychological adjustment in the presence of prolonged stressors.

In conclusion, the present longitudinal study sheds light on positive and negative mental health variables in key phases of the COVID-19 pandemic. Overall, the combined results point out that: (1) individuals experienced significant negative effects on their mental health after almost a year of the pandemic, both on ED and on the positive functioning variables; (2) this adverse situation also contributed to the development of certain dimensions of PTG over time, but it negatively affected relations with others; (3) resilience scores measured in different phases of the COVID-19 crisis were constant predictors of positive mental health, openness to the future, and burden experienced during the third wave of infections; and (4) the effect of resilience on psychological burden was explained by the mediating role of positive mental health and openness to the future. Overall, our results suggest that the Spanish population experienced significantly negative effects on their mental health due to the prolonged duration of the pandemic, but at the same time, they developed some coping strategies that led to growth. In sum, depending on the moment, the COVID-19 pandemic allowed Spanish citizens to make lemonade from the metaphorically served lemons, whereas at other times, the taste might have seemed too sour.

## Data Availability

The datasets generated and/or analyzed during the current study are available in the OSF repository:https://osf.io/upnyx/.
